# Perception of Risk and Terrorism-Related Behavior Change: Dual Influences of Probabilistic Reasoning and Reality Testing

**DOI:** 10.3389/fpsyg.2017.01721

**Published:** 2017-10-05

**Authors:** Andrew Denovan, Neil Dagnall, Kenneth Drinkwater, Andrew Parker, Peter Clough

**Affiliations:** ^1^Department of Psychology, Manchester Metropolitan University, Manchester, United Kingdom; ^2^Department of Psychology, University of Huddersfield, Huddersfield, United Kingdom

**Keywords:** perception of risk, probabilistic reasoning, reality testing, terrorism-related behavior change, thinking style

## Abstract

The present study assessed the degree to which probabilistic reasoning performance and thinking style influenced perception of risk and self-reported levels of terrorism-related behavior change. A sample of 263 respondents, recruited via convenience sampling, completed a series of measures comprising probabilistic reasoning tasks (perception of randomness, base rate, probability, and conjunction fallacy), the Reality Testing subscale of the Inventory of Personality Organization (IPO-RT), the Domain-Specific Risk-Taking Scale, and a terrorism-related behavior change scale. Structural equation modeling examined three progressive models. Firstly, the Independence Model assumed that probabilistic reasoning, perception of risk and reality testing independently predicted terrorism-related behavior change. Secondly, the Mediation Model supposed that probabilistic reasoning and reality testing correlated, and indirectly predicted terrorism-related behavior change through perception of risk. Lastly, the Dual-Influence Model proposed that probabilistic reasoning indirectly predicted terrorism-related behavior change via perception of risk, independent of reality testing. Results indicated that performance on probabilistic reasoning tasks most strongly predicted perception of risk, and preference for an intuitive thinking style (measured by the IPO-RT) best explained terrorism-related behavior change. The combination of perception of risk with probabilistic reasoning ability in the Dual-Influence Model enhanced the predictive power of the analytical-rational route, with conjunction fallacy having a significant indirect effect on terrorism-related behavior change via perception of risk. The Dual-Influence Model possessed superior fit and reported similar predictive relations between intuitive-experiential and analytical-rational routes and terrorism-related behavior change. The discussion critically examines these findings in relation to dual-processing frameworks. This includes considering the limitations of current operationalisations and recommendations for future research that align outcomes and subsequent work more closely to specific dual-process models.

## Introduction

Acts of terrorism result in tens of thousands of deaths annually (Institute for Economics and Peace, 2016) and negatively affect people worldwide (Hobfoll et al., 2007). Consequently, mass media outlets prominently feature news of terrorist attacks, threats and risks (domestic and international). This coverage reflects the fact that terrorist incidents/activity are a major cause of concern within contemporary societies (Goodwin et al., 2005). Indeed, perceived risk of terrorist attack is so pervasive that it can instigate behavior change, including lifestyle choices (Torabi and Seo, 2004), transportation preference, (Blalock et al., 2009), travel intentions, (Reisinger and Mavondo, 2005) and selection of tourist destinations (Sönmez and Graefe, 1998). Relatedly, previous research indicates that perceived risk has a moderate positive direct and indirect (mediating) effect on behavior change in response to perceived terrorist threat (Goodwin et al., 2005). Specifically, Goodwin et al. (2005) established that greater perceived risk informed a reduction in planned air travel, use of public transport, and changes to daily routine, with risk additionally determining behavior change in response to individual differences (age, gender, personality).

At a societal and individual level, terrorist activity reinforces anxieties, heightens awareness of potential risk and increases the likelihood of further behavior change (Finseraas and Listhaug, 2013). Behavior change ensues when appraisal of risk (likelihood and severity) exceeds perceived ability to cope (Rogers, 1983, 1985). Once this occurs, protection motivation (the desire to perform protective activities) is aroused and behavioral intentions and attitudes alter (Rogers, 1975, 1983; Qi et al., 2009). In this context, perception of risk has potentially profound effects on individual and collective actions.

The current paper contends that thinking style is an important factor likely to influence perception of risk and terrorism-related behavior change. Thinking style embodies trait-like differences in information processing related to two extensively researched modes of thinking, objective-rational vs. subjective-intuitive (Kozhevnikov, 2007). The former employs logic and appreciation of probability to guide decision and opinion outcomes, whilst the latter bases decision making on intuition and affective appraisal of situations/events, and is experiential (Epstein, 2003). Acknowledging this crucial distinction, the present paper operationalised objective-rational thinking style in terms of probabilistic reasoning ability and subjective-intuitive thinking style in relation to proneness to reality testing deficits. The degree to which these ‘thinking styles’ influenced perception of risk and terrorism-related behavior change was then assessed. A delineation of these concepts follows discussion of the characteristics of risk perception.

### Risk Perception

Risk is a psychological concept based on individual perception rather than empirical fact (Slovic and Weber, 2015). Threat-related inference derives from personal evaluation of qualitative characteristics related to a situation/stimulus, rather than objective ‘hazard’ features (Slovic, 1987). Personal judgment determines which information defines danger even in circumstances where level of risk is evident and associated probabilities known (Jenkin, 2006).

Misperception of risk is important because it is likely to produce ineffective or even detrimental behavior adaptation. Illustratively, the 9/11 terrorist attacks facilitated migration from flying to driving (Blalock et al., 2009). A behavioral change that paradoxically increased driving associated deaths (estimated at 2170). This mismatch between risk (actual vs. perceived) and behavior change could arise for several reasons. For instance, misappreciation of hazard likelihood or bounded rationality, where factors other than estimation of probability determine decision-making (cf. Gray and Ropeik, 2002; Kahneman, 2003; Cokely and Kelley, 2009).

### Factors Influencing Risk Perception

This study assessed the extent to which variables, previously identified as important in guiding judgments under uncertainly, influenced decision making related to potential threat. The first factor was probabilistic reasoning ability. This was conceptualized in terms of a thinking style involving rational appraisal of probabilistic outcomes (Denes-Raj and Epstein, 1994; Epstein et al., 1999). The general defining feature of this rational approach is a focus on objective evaluation of environmental features.

Consequently, the rational thinking style is measurable via probabilistic reasoning tasks. Performance provides an indication of the degree to which individuals make accurate use of statistical information in decision-making situations. This approach is germane to the study of risk perception (c.f. Slovic and Weber, 2015) and is rooted in the decision-making tradition of Kahneman and Tversky (e.g., Kahneman and Tversky, 1982; Tversky and Kahneman, 1982). In tandem, probabilistic measures assessed the extent to which thinking style informed perception of risk (Schwenk, 1984) and self-reported terrorism-related behavior change. Concomitant research in the domain of unconventional beliefs has revealed that ‘apparently’ related anomalous beliefs (paranormal, conspiracies and urban legends) are associated with important cognitive-perceptual processing differences (Dagnall et al., 2016a,b).

The second factor, related to outcome judgment was subjective evaluation. This derives largely from self-generated cognitions, perceptions and interpretations, such as intuition and personal experience. The present study used the reality testing subscale of the Inventory of Personality Organization (IPO-RT) (Lenzenweger et al., 2001) to index this thinking style. The IPO-RT assesses the inclination to intuitive-experiential (vs. analytical-rational) processing. Specifically, “the capacity to differentiate self from non-self, intra psychic from external stimuli, and to maintain empathy with ordinary social criteria of reality” (Kernberg, 1996, p. 120). Principally, the IPO-RT assesses subjective propensity to reality testing errors via the ability to distinguish between internal and external sources. Accordingly, the researchers reasoned that suspension of reality testing, or proneness to reality testing deficits reflected a preference for an intuitive-experiential information processing style (Irwin, 2004).

From a broader perspective, these variables can be conceptualized as risk perception deriving from an objective analysis of the situation ‘risk as analyses,’ and from a more intuitive feeling based assessment ‘risk as feelings’ (Slovic and Peters, 2006). The former, refers to judgment of risk arising using scientific deliberation, logic and reason. From this perspective, perception of danger arises as a function of the systematic appraisal of threat-related information, and the utilization of cognitive strategies and algorithmic rules that allow for an effective combination of decision elements into an overall judgment. This deliberate analytical process influences behavior change through appraisal-based elements, including the weighing up of potential risk (Epstein et al., 1996). Contrastingly, risk as feelings draws upon intuitive reactions that are the outcome of fast associative processing based on experience. This process is guided by heuristics and biased by affective signals (Slovic et al., 2004), self-efficacy (Stevens et al., 2012) and shared normative perceptions (Goodwin et al., 2005). A predominant characteristic of risk as feelings is the influence of affective signals and the motivated behavior that results in order to prolong or avoid experiences.

### The Current Study

Previous work suggests that, although positively correlated, general perception of risk and terrorism-related behavior originate from different thought processes. Specifically, cognitive evaluations (risk as analyses) influence perception of risk, whilst affect (risk as feelings) promotes terrorism-related behavior change. Indeed, a significant body of knowledge designates that incorrect perception of risk stems from faulty estimation of real world danger. For example, risk related to dramatic or sensational causes of death (e.g., accidents, homicides, cancer and natural disasters) is often overestimated and risk associated with conventional/mundane sources (e.g., common illness) under-estimated (Slovic et al., 1979; Slovic, 2016). These findings suggest a strong relationship between perception of risk and probabilistic reasoning performance. Contrastingly, terrorism-related behavior change because of its affective element should relate more strongly to an intuitive thinking style. This view accords with theoretical conceptualizations of terrorism, which depict terrorism as acts and actions intended to impart fear and create feelings of vulnerability and insecurity (Gross et al., 2016). In addition, previous research indicates that perception of risk has a direct and indirect effect on terrorism-related behavior change (Goodwin et al., 2005); this should occur specifically in relation to probabilistic reasoning (Epstein et al., 1996).

**FIGURE 1:**
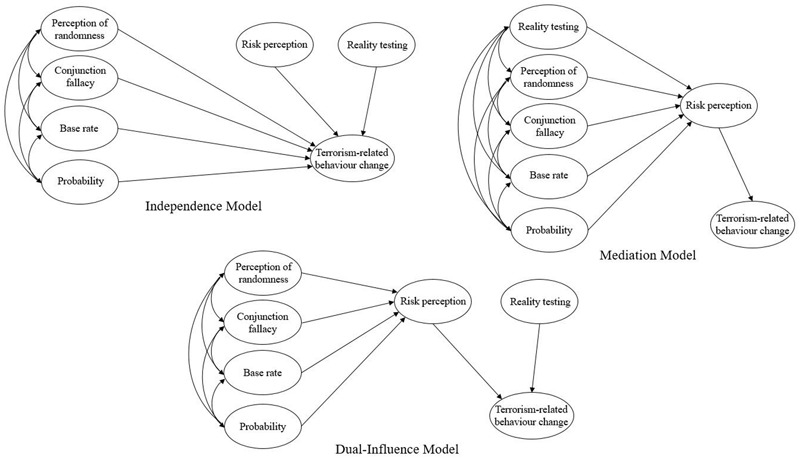
Competing models of the potential relationships among probabilistic reasoning, reality testing, risk perception, and terrorism-related behavior change.

The present study used structural equation modeling (SEM) to examine the dual-influence of probabilistic reasoning (rational) vs. reality testing (intuitive) in relation to risk perception and terrorism-related behavior change. Three different theoretically driven models were tested (**Figure 1**). The first, Independence Model, was parsimonious and conceptually simple. It assumed that no relationships existed between probabilistic reasoning, risk perception and reality testing, and that each of these constructs independently predicted terrorism-related behavior change. The second, Mediation Model, supposed that probabilistic reasoning and reality testing correlated, and had an indirect (mediated) effect on behavior change through risk perception. The third, Dual-Influence Model, proposed that probabilistic reasoning (analytical-rational processing), indirectly predicted terrorism-related behavior change via perception of risk, independent of reality testing (intuitive-experiential processing). Collectively, these models represented the most plausible theoretical solutions. The method of comparing theoretically acceptable models facilitates better comprehension of inter-relationships among factors. This approach has proved highly successful within other areas of psychology (see Hubbard and Mannell, 2001).

## Materials and Methods

### Participants

A sample of 263 participants (139 women, 53% and 124 men, 47%) took part in this study. The mean age was 32.50 (*SD* = 14.16), and the age range was 18–87 years. The mean age for men was 31.38 (*SD* = 13.38; range = 18–77 years), and the mean age for women was 33.50 (*SD* = 14.56; range = 18–87 years). Respondents included undergraduates, postgraduates and employees from Manchester Metropolitan University (MMU) together with members of the wider community. Recruitment was via social media, university staff email, and through local stakeholders (businesses, and vocational classes). Involvement was voluntary and responses anonymised. Participants could withdraw up to 4 weeks after data collection. Exclusion criteria required that respondents were at least 18 years of age, had not previously studied heuristic bias and had not taken part in previous probabilistic reasoning research.

### Materials

### Domain-Specific Risk-Taking Scale (DOSPERT)

The revised DOSPERT (Weber et al., 2002; Blais and Weber, 2006) assessed general perception of risk. The measure contains 30-items assessing behavioral intentions, the degree to which respondents believe they are likely to engage in risky behaviors. Items appear as statements and relate to five domains of life (ethical, financial, health/safety, social and recreational risks). For example, ‘piloting a small plane.’ Respondents indicate their perceived level of risk for each situation on a 7-point scale; 1 = not at all risky to 7 = extremely risky. Total scores range from 30 to 210, with upper scores indicating higher levels of perceived risk. The DOSPERT has been widely used within research and is a validated scale, possessing established psychometric properties. The original 40-item DOSPERT scale demonstrated satisfactory internal consistency, adequate test–retest reliability and validity (Weber et al., 2002), and in this study the DOSPERT was internally consistent (*α* = 0.85).

#### Behavior Change

Behavior change items developed by Sinclair and LoCicero (2006) assessed the degree to which respondents perceived their behavior had changed because of previous terrorist activity. Items originated from open-ended statements presented to focus groups (Sinclair and LoCicero, 2006). Questions asked participants about behavior change since 9/11. Emergent items related to common aspects of everyday life (fear of flying, avoiding cities, public transportation, etc.). In the present study, statements were adapted for use with a UK sample. For instance, ‘trams’ replaced ‘subways’ and ‘shopping centers’ replaced ‘malls.’ To avoid offense to potential participants the researchers omitted a statement referring to interacting with Middle Eastern or people of Arab descent. Thus, the modified behavior change measure comprised eight-items. Responses were recorded on a Likert scale ranging from 1 (I do it about the same) to 5 (I do not do it anymore); total scores ranged from 8 to 40. Higher scores indicated increased levels of terrorism-related behavior change. Previous work found the original behavior change items functioned as a single construct possessing good psychometric properties (internal reliability, face validity and conceptual similarity) (Sinclair and LoCicero, 2006). In this study, the terrorism-related behavior change measure demonstrated good internal reliability (*α* = 0.73).

#### Reality Testing Inventory of Personality Organization

The reality testing subscale of the Inventory of Personality Organization (IPO-RT) (Lenzenweger et al., 2001) assessed preference for intuitive (vs. rational) thinking. The IPO-RT is a unidimensional self-report measure, which measures ‘the capacity to differentiate self from non-self, intrapsychic from external stimuli and to maintain empathy with ordinary social criteria of reality’ (Kernberg, 1996, p. 120). Accordingly, the IPO-RT focuses on information processing style rather than psychotic symptomology (Langdon and Coltheart, 2000; Irwin, 2004). The IPO-RT comprises 20 statements (e.g., ‘I feel that my wishes or thoughts will come true as if by magic’). Participants respond via a five-point Likert scale (1 = never true to 5 = always true). Total scores range from 20 to 100, with low scores indicating high reality testing (preference for analytical-rational thinking). Several recent studies have used the IPO-RT because it possesses good psychometric properties. Lenzenweger et al. (2001) report the scale is internally consistent and temporally stable with non-clinical populations. Indeed, the IPO-RT demonstrates retest reliability (*r* = 0.73) and good construct validity (Lenzenweger et al., 2001). The IPO-RT possessed excellent internal reliability in this study (*α* = 0.91).

#### Probabilistic Reasoning Tasks

Twenty tasks assessed appreciation of probabilistic reasoning. These problems have featured in several previous studies (Dagnall et al., 2007, 2014). Tasks were organized into four sections comprising one of each probabilistic reasoning type (perception of randomness, base rate, probability and conjunction). Responses required that participants selected one answer (the most likely outcome) from a range of alternatives. Tasks function as independent measures of probabilistic reasoning performance (scored 0–5) and provide an overall measure of probabilistic reasoning ability (scored 0–20). In all instances, higher scores indicate greater appreciation of probabilistic reasoning. The tasks included were:

##### Perception of randomness

This required participants to judge the likelihood of obtaining various strings/sequences (e.g., ‘when tossing a coin six times, which pattern of results do you think is most likely? (a) HHHHHH, (b) HHHTTT, (c) HTHHTT, (d) all equally likely’).

##### Base rate

Participants evaluated the outcome of probability based on presented base rate evidence (e.g., ‘you go to a party where there are 100 men, 70 of the men are psychologists and 30 are engineers. Prior to meeting a man, the host provides a short personality description: Jack is a 45-year old man. He is married with four children. He is generally conservative, careful and ambitious. He shows no interest in politics or social issues and spends much of his free time on his hobbies, which include carpentry, sailing and mathematical puzzles. What is the probability that Jack is an engineer? (a) 100%, (b) 70%, (c) 50%, (d) 30%’).

##### Probability

Participants selected the correct likelihood of success from alternative scenarios (e.g., ‘Melissa shuffled a deck of number cards containing five each of the numbers two, four, six, and seven. If Melissa randomly selects a four from the deck and does not return it, what is the probability that she will select a four on her next draw? (a) 3/20 [0.15], (b) 4/5 [0.80], (c) 4/19 [0.21], (d) 1/4 [0.25]’).

##### Conjunction fallacy

From a range of events, participants specified the most likely outcome. Statements were presented either as single or co-occurring events (e.g., ‘two football teams [Team A and Team B] are playing in a local derby. What is the most likely outcome of the game?’ (a) Team A score first and the game is drawn, (b) Team A score first and win, (c) Team A score first and lose, (d) Team A scores first’). Within conjunction problems, the likelihood of event intersection could not exceed the probability of single constituent events (c.f., Tversky and Kahneman, 1982, 1983).

### Procedure

Prior to involvement, potential participants read the background information. This stated that the research was concerned with cognitive-perceptual personality factors related to perceptions of risk and terrorism. Only participants consenting to take part received the materials booklet. Instructions within the booklet asked participants to take their time and to answer questions openly and honestly. The booklet comprised five subsections: demographic information (completed first), reality testing, risk perception, terrorism-related behavior change and probabilistic reasoning tasks. To avoid order effects, section sequence rotated across respondents.

### Ethics Statement

The researchers obtained ethical approval for the study as part of a research proposal examining relationships between anomalous beliefs and cognitive-perceptual measures (‘Statistical Bias, Context and Anomalous Beliefs: A Critical Evaluation’). The Director of the Research Institute for Health and Social Change located within the Faculty of Health, Psychology and Social Care within MMU approved the project (methodological and ethical); 01/08/2017. This is the necessary level of ethical clearance for projects rated as ‘routine.’ It is a university condition that all proposals are peer-reviewed by members of the Professoriate (or equivalent) prior to submission. This includes ethical scrutiny and gaining clearance in principle. Additionally, the Head of the Psychology Department must sanction research projects. Respondents indicated informed consent by ticking a box prior to participation; no personal information was collected other than age, preferred gender and general location. This procedure ensured that respondents had read and understood the instructions. Formal submission to a university ethics panel beyond this process is not an institutional requirement for routine studies.

### Analytical Strategy

Analysis involved two related phases. The first examined variable means, standard deviations and zero-order correlations (see **Tables 1–3**). The second phase employed structural equation modeling (SEM) using AMOS 23 to examine the fit of three competing models. Model 1 (Independence Model) assumed that probabilistic reasoning, risk perception and reality testing independently predicted terrorism-related behavior change. Model 2 (Mediation Model) presumed that probabilistic reasoning and reality testing had an indirect (mediated) effect on terrorism-related behavior change through risk perception. Within SEM, mediation analysis examines whether specific variables act as an intermediary between other variables. In the present study, this was risk perception. Mediation analysis within psychology is important because it reveals mechanisms underlying the relationships between variables. Model 3 (Dual-Influence Model) anticipated that reality testing predicted terrorism-related behavior change independent of the probabilistic reasoning-risk perception route, and that probabilistic reasoning had an indirect effect through risk perception on terrorism-related behavior change.

**Table 1:** Descriptive information and intercorrelations among reality testing, terrorism-related behavior change, risk perception, and risk perception subscales.

	*M*	*SD*	1	2	3	4	5	6	7	8
(1) Reality testing	35.77	11.18								
(2) Behavior change	12.05	4.15	0.27**							
(3) Perception of risk total	130.44	20.97	0.11	0.26**						
(4) Ethical risk	28.53	5.95	0.03	0.20**	0.78**					
(5) Financial risk	28.49	5.90	0.06	0.14*	0.67**	0.38**				
(6) Health/Safety risk	30.54	5.92	-0.01	0.20**	0.79**	0.64**	0.38**			
(7) Recreational risk	25.20	6.45	0.09	0.25**	0.79**	0.48**	0.42**	0.50**		
(8) Social risk	17.67	4.97	0.24**	0.11	0.53**	0.25**	0.18**	0.25**	0.34**	

*^∗^*p* < 0.05, ^∗∗^*p* < 0.001.*

**Table 2:** Descriptive information and intercorrelations among probabilistic reasoning tasks.

	*M*	*SD*	*Proportion*	1	2	3	4	5
(1) Perception of randomness	3.90	1.06	77.19					
(2) Base rate	1.74	0.96	34.68	0.24^∗∗^				
(3) Conjunction fallacy	2.17	1.40	43.65	0.36^∗∗^	0.27^∗∗^			
(4) Probability	2.39	1.04	47.30	0.24^∗∗^	0.21^∗∗^	0.29^∗∗^		
(5) Overall problem total	10.21	3.03	50.70	0.67^∗∗^	0.60^∗∗^	0.77^∗∗^	0.63^∗∗^	

*^∗^*p* < 0.05, ^∗∗^*p* < 0.001.*

**Table 3:** Zero-order correlations between probabilistic reasoning tasks, reality testing, risk perception and terrorism-related behavior change.

	Risk perception	Behavior change	Reality testing
Perception of randomness	-0.08	-0.13^∗^	-0.09
Base rate	-0.30^∗∗^	-0.14^∗^	-0.06
Conjunction fallacy	-0.27^∗∗^	-0.18^∗^	-0.05
Probability	-0.17^∗^	-0.19^∗^	-0.10
Overall problem total	-0.31^∗∗^	-0.24^∗∗^	-0.11

*^∗^*p* < 0.05, ^∗∗^*p* < 0.001.*

Several fit indices assessed relative model fit. These comprised measures of absolute and incremental fit. Absolute indices (i.e., Standardized Root Mean Square Residual, SRMR and Root-Mean-Square Error of Approximation, RMSEA) determine how well *a priori* models fit the sample data and indicate which proposed model possesses superior fit (Hooper et al., 2008). Incremental indices (i.e., the Comparative Fit Index, CFI and the Incremental Fit Index, IFI) compare observed chi-square values with baseline models and assume that all latent variables are uncorrelated (null model) (Hooper et al., 2008).

An acceptable model requires SRMR < 0.08, RMSEA < 0.08, CFI > 0.90 and IFI > 0.90 (Brown and Cudeck, 1993). Marginal fit is indicated by SRMR and RMSEA values of 0.08–0.10 and CFI and IFI values of 0.86–0.90 (Nigg et al., 2009). RMSEA used the 90% confidence interval. Bootstrapping estimates (resampled 5000 times using the bias-corrected percentile method to create 95% confidence intervals) examined indirect effects for the Mediation and Dual-Process Models. The Akaike Information Criterion (AIC) and Expected Cross-Validation Index (ECVI), both measures of quality of statistical models, compared non-nested models. Lower values indicated superior fit.

## Results

### Preliminary Analyses

Assumptions were initially checked (outliers, multicollinearity, linearity), and eight participants were removed due to possessing *z*-scores greater than 3.29 or less than -3.29 (Tabachnick and Fidell, 2007) leaving a final sample of 255. Following data screening, means, standard deviations and bivariate correlations for scales were calculated. Perception of risk positively correlated with terrorism-related behavior change, *r*(253) = 0.26, *p* < 0.001, and terrorism-related behavior change positively correlated with reality testing, *r*(253) = 0.27, *p* < 0.001. In addition, ethical, recreational, financial, and health/safety risk factors positively correlated with terrorism-related behavior change (see **Table 1**). There was no significant association between perception of risk and reality testing, *r*(253) = 0.11, *p* = 0.095.

Descriptive statistics for probabilistic reasoning tasks together with intercorrelations appear in **Table 2**. Probabilistic reasoning tasks correlated positively, relationships were in the moderate range (ranged from *r* = 0.21 to *r* = 0.36).

Correlations between probabilistic reasoning tasks, reality testing, risk and behavior change appear in **Table 3**. Results indicated that terrorism-related behavior change was significantly negatively associated with all indicators of probabilistic reasoning (perception of randomness, conjunction fallacy, base rate and probability). Risk perception significantly negatively correlated with all probabilistic reasoning types, except perception of randomness. Reality testing only weakly correlated with probability. Observing that perception of randomness was weakly associated with risk-related factors, subsequent analysis excluded perception of randomness (perception of randomness failed to correlate with risk perception and was only weakly associated with terrorism-related behavior change).

### Measurement Model

The first step in model evaluation was to examine the measurement model. This depicted latent variables as covarying (probabilistic reasoning, reality testing, risk perception and behavior change). In order to increase degrees of freedom and the statistical power of the tested models (Coffman and MacCallum, 2005), item parceling within latent variables occurred. Additionally, item parcels are more likely to be normally distributed, and to meet the assumptions of the maximum likelihood method typically used in confirmatory factor analysis and SEM (Thompson and Melancon, 1996). Exploratory factor analysis (EFA) using oblique (promax) rotation assessed items on each latent variable. The observed factor loadings informed item to parcel allocation, with apportionment corresponding to descending order (Coffman and MacCallum, 2005).

EFA reported that each indicator of probabilistic reasoning possessed a single factor structure. In order to include these factors as latent variables, variance was determined by multiplying scale variance with alpha reliability (Kline, 2011). EFA revealed reality testing comprised four factors and terrorism-related behavior change comprised two factors. Both of these measures were subjected to EFA because inconsistency exists regarding the definitive factor structure for the reality testing subscale of the IPO-RT (Ellison and Levy, 2012), and because the behavior change measure was adapted for use within the current study. Byrne (2013) advocates EFA when no strong conceptual underpinning is present. The four-factor solution for reality testing (IPO-RT) accounted for 56.2% of variance (all loadings > 0.4 apart from items 17 and 4). Factor 1 (items 2, 5, 7, 8, 9, 16, 17) was labeled ‘auditory and visual hallucinations’; factor 2 (items 12, 14, 15, 18, 19) was labeled ‘delusional thinking’; factor 3 (items 4, 10, 13, 20) was labeled ‘social deficits’; factor 4 (items 1, 3, 6, 11) was labeled ‘confusion.’ Identification of factors was consistent with theoretical underpinnings of reality testing deficits and previous factor analyses of the reality testing subscale of the IPO-RT (Bell et al., 1985; Caligor and Clarkin, 2010; Dagnall et al., 2017). The two-factor solution for terrorism-related behavior change accounted for 55.3% of variance, with all variables (apart from item 4) loading > 0.4. The factors were labeled as ‘changes to travel habits’ (items 2, 1, 3), and ‘changes to personal preferences’ (items 6, 7, 5, 8, 4). Existing subscales (ethical, financial, health/safety, social and recreational risks) determined the risk perception latent variable. Established research supports the hierarchical factor structure of this measure (Blais and Weber, 2006). The measurement model demonstrated acceptable fit, *χ^2^*(65, *N* = 255) = 109.38, *p* < 0.001, CFI = 0.95, TLI = 0.92, SRMR = 0.05, RMSEA = 0.05 (CI of 0.03–0.06), and all factor loadings were significant (*p* < 0.001).

### Structural Models

The Independence Model (Supplementary Figure 1) demonstrated acceptable data-model fit on all criteria, but SRMR, *χ^2^*(72, *N* = 255) = 147.64, *p* < 0.001, CFI = 0.91, IFI = 0.91, SRMR = 0.09, RMSEA = 0.06 (CI of 0.04–0.07). SRMR revealed marginal fit. Structural paths indicated that probabilistic reasoning did not have a significant negative effect on terrorism-related behavior change. However, it is important to note that direct effects are not a necessary condition to test for mediation in the Mediation and Dual-Influence Models. Instead, emphasis should be on testing the magnitude of indirect effects (for a discussion see Rucker et al., 2011). Risk perception had a significant positive effect on terrorism-related behavior change (*β* = 0.31, *p* = 0.022), as did reality testing (*β* = 0.34, *p* < 0.001). Overall, the Independence Model accounted for 29% of the variance in behavior change.

The Mediation Model (Supplementary Figure 2) possessed acceptable fit on all indices, *χ^2^*(69, *N* = 255) = 128.80, *p* < 0.001, CFI = 0.93, IFI = 0.93, SRMR = 0.07, RMSEA = 0.05 (CI of 0.04–0.07). The structural paths indicated that of the probabilistic reasoning types, conjunction fallacy had a significant negative effect on risk perception (*β* = -0.41, *p* = 0.030). Reality testing did not have a significant effect on risk perception (*β* = 0.04, *p* = 0.630) and possessed a non-significant correlation with all indicators of probabilistic reasoning. Risk perception, meanwhile, had a significant positive effect on terrorism-related behavior change (*β* = 0.40, *p* < 0.001). Bootstrapping estimates indicated that conjunction fallacy had an indirect effect on terrorism-related behavior change through risk perception that was significant at the 95% confidence level across bias-corrected percentile point estimates (*p* = 0.021, 95% Cl = -0.41 to -0.03). Specifically, the standardized indirect effect was -0.16, suggesting that behavior change decreases by 0.16 standard deviations for every unit increase in conjunction fallacy indirectly through risk perception. The model accounted for 26% of the variance in risk perception and 16% of the variance in terrorism-related behavior change.

**FIGURE 2:**
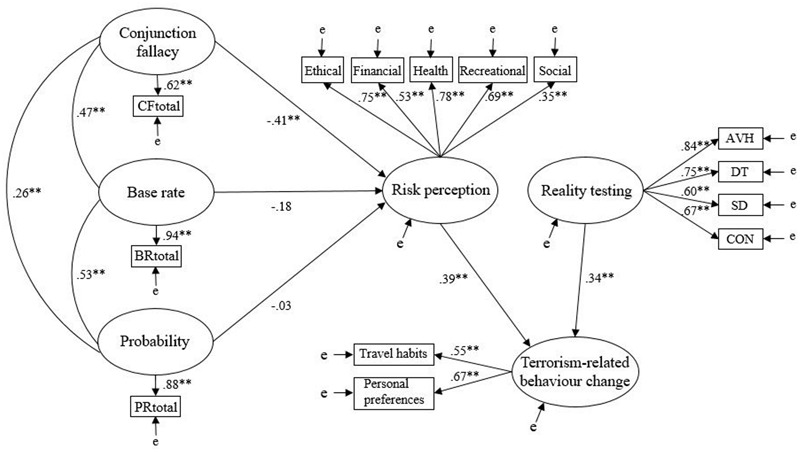
Dual-Influence Model. AVH, auditory and visual hallucinations; DT, delusional thinking; SD, social deficits; CON, confusion. Latent variables are represented by ellipses; observed variables are represented by rectangles; error of measurement is indicated by ‘e’; ^∗^*p* < 0.05, ^∗∗^*p* < 0.001.

The Dual-Influence Model (**Figure 2**) demonstrated acceptable fit on all indices, *χ^2^*(72, *N* = 255) = 117.89, *p* = 0.001, CFI = 0.94, IFI = 0.95, SRMR = 0.06, RMSEA = 0.05 (CI of 0.03–0.06). Consistent with the Mediation Model, conjunction fallacy had a significant negative effect on risk perception (*β* = -0.41, *p* = 0.030), and risk perception had a significant positive effect on terrorism-related behavior change (*β* = 0.39, *p* < 0.001). In addition, reality testing had a significant positive effect on terrorism-related behavior change (*β* = 0.34, *p* < 0.001). Bootstrapping estimates indicated that conjunction fallacy had a significant indirect effect on terrorism-related behavior change through risk perception across bias-corrected percentile point estimates (*p* = 0.014, 95% Cl = -0.41 to -0.03). The standardized indirect effect was -0.16, and the model explained 25% of the variance in risk perception and 27% of the variance in behavior change. A comparison of AIC and ECVI values revealed that the Dual-Influence Model (AIC = 211.89; ECVI = 0.83) provided a superior data fit than the Independence Model (AIC = 241.64; ECVI = 0.95) and the Mediation Model (AIC = 228.80; ECVI = 0.90).

Overall, conjunction fallacy had a significant indirect effect on terrorism-related behavior change via risk perception. These findings specified an analytical-rational route to behavior change. Results also indicated that reality testing does not directly predict risk perception, but independently predicts behavior change supporting an intuitive-experiential route to terrorism-related change behavior as a function of perceived threat.

## Discussion

### Consideration of the Current Findings

As hypothesized, scores on probabilistic reasoning tasks most strongly predicted perception of risk, and preference for an intuitive thinking style (as indexed by IPO-RT) best explained terrorism-related behavior change. The combination of risk with indicators of probabilistic reasoning in the Dual-Influence Model enhanced the predictive power of the rational-analytical route. Consequently, the Dual-Influence Model found similar predictive relations between the intuitive and analytical thinking styles.

With regard to perception of risk, lower performance on probabilistic reasoning tasks was moderately associated with higher levels of risk perception and weakly correlated with increased terrorism-related behavior change. Contrastingly, the association between reality testing and terrorism-related behavior change was in the weak to moderate range. Additionally, there was no meaningful correlation between reality testing and risk perception.

Perception of risk was negatively associated with performance on probabilistic reasoning tasks. Furthermore, conjunction fallacy predicted behavior change via risk perception (mediated). Indeed, conjunction fallacy had a negative impact on risk. The finding that poorer appreciation of probabilistic information predicted higher perception of risk is consistent with earlier work that demonstrated inaccurate perception of risk arises from a weak understanding of event likelihood (Slovic et al., 1979; Slovic, 2016). In addition, Koblentz (2011) reported that proneness to conjunction error negatively influenced the ability to estimate accurately terrorism-related risk.

In turn, the results of this study indicate that a lower propensity to make conjunction errors has a positive impact on risk perception, with risk perception predicting a greater likelihood of behavior change. These results support the notion that probabilistic bias (particularly conjunction error) has the potential to lead to a misperception of risk, which is likely to produce ineffective or even detrimental behavior adaptation in response to terrorism-related threat (Blalock et al., 2009). With regard to risk perception and specific forms of probabilistic reasoning bias, it is important to acknowledge that multiple heuristics may operate within situations. Hence, the current finding regarding conjunction could reflect the perception of risk used (Peters et al., 2006). Indeed, preceding research reports that task framing influences the likelihood of conjunction error (Nilsson et al., 2013). In terms of risk, conjunction may occur via the availability heuristic, whereby threat assessments are associated with recent negative experiences or events producing the risk (Peters et al., 2006). In the case of conjunction, this may involve referencing risk to specific threats and consequences. Considering conjunction fallacy more generally, it is conceptually unclear which heuristics facilitate conjunction error. For instance, Tversky and Kahneman (1983), in their important formative article, contended the representativeness heuristic caused conjunction error. However, a body of papers since have demonstrated that the representativeness heuristic is far from a necessity for the conjunction error to occur (Gavanski and Roskos-Ewoldsen, 1991; Nilsson, 2008; Nilsson et al., 2013). The finding from this study that risk perception had a moderate positive effect on terrorism-related behavior change is consistent with previous work (Goodwin et al., 2005), supporting the notion that an increased sense of threat has a key role concerning terrorism-related behavior change likelihood.

With regard to risk scores, DOSPERT indicated moderate levels of engagement with risky behaviors. This was not surprising because the measure samples common behaviors across several key life domains (ethical, financial, health/safety, social and recreational risks). Collectively, the DOSPERT provides a broad overview of risk, however, the degree to which risk influences terrorism-related behavior change may vary as a function of domain and risk type. Indeed, ethical, health/safety, financial, and recreational risk factors correlated positively with increased terrorism-related behavior change. The strongest relationships existed for health/safety, recreational, and ethical. The findings for health/safety and recreational make sense given that key aspects of terrorism-related behavior change relate to lifestyle choices (Torabi and Seo, 2004). The finding for ethical is less clear-cut, but it is possible that this outcome pertains to a greater awareness of order, rules, and increased vigilance, which Stevens et al. (2012) found was a commonly reported behavioral response to a perceived terrorist threat.

Reported levels of terrorism-related behavior change were small and attributable mainly to items connected to travel habits (using public transportation, flying on commercial airplanes) and personal preferences (avoidance of cities, voting behavior). This aligned with preceding work identifying terrorism-related behavior adaptions in attendant areas of everyday life (e.g., lifestyle choices, Torabi and Seo, 2004; mode of transportation, Blalock et al., 2009; travel intentions, Reisinger and Mavondo, 2005; and selection of tourist destinations, Sönmez and Graefe, 1998).

### Broader Theoretical Context

Dual-process theories of cognition (Sloman, 1996; Chaiken and Trope, 1999; Kahneman and Frederick, 2002) provide a useful conceptual framework for interpreting and contextualizing the current findings. However, the range, complexity and diversity of dual-processing theories limits the formation of precise conclusions (Evans, 2008). Hence, extrapolations within the present paper appeal to the dual-processing approach generally, rather than specific models and processes.

In this context, the dual-processing approach proposes that judgment outcomes arise from two distinct, but interrelated systems (cognitive evaluations vs. emotions) (Loewenstein et al., 2001). Cognitive evaluations utilize effortful, intentional, attention demanding processes and base decisions on analytical appraisal of information. Thus, cognitive evaluations use established rules of logic and consider empirical evidence (Slovic et al., 2004). Contrastingly, emotions derive from affect and use general cognitive heuristics. A related dual-process theory is Cognitive–Experiential Self-Theory (CEST, Epstein, 1994). A key feature of CEST is its focus on ‘thinking or cognitive style.’ Specifically, CEST differentiates between rational (controlled) and experiential (automatic) processing. The rational system is slow, effortful and demanding of attention, whilst the intuitive system is automatic, fast and non-conscious (Epstein, 2003). Each thinking style directs information processing (Kozhevnikov, 2007).

From a dual-processing perspective, this study indicated that different processes might influence perception of risk and terrorism-related behavior change. This is a useful conceptual distinction for future work to elaborate and assess. Particularly, the notion that perception of risk arose from effortful cognitive evaluations (risk as analyses), whilst terrorism-related behavior change derived from subjective-personalized thinking (risk as feelings) (Loewenstein et al., 2001). These suppositions are congruent with the CEST model. Particularly, the view that terrorism-related behavior change develops more from feeling, emotion and intuition and less from analytic cognition (Epstein, 1994).

Indeed, acts of terrorism by their inherent nature are typically dramatic and violent in content and evoke emotional reactions likely to facilitate experiential processing. Resultant responses should then bias people away from consideration of the actual or more objectively defined risk. This may occur because emotions provide a rapid, efficient basis for directing behavior in complex, uncertain real-world situations (Zajonc, 1980; Slovic and Peters, 2006). From this viewpoint, emphasis on deriving judgments based on intuition and emotion reduces the relative importance of empirical fact-based information (see below).

Similarly, the finding that combining risk perception with probabilistic reasoning enhanced the predictive power of the cognitive-analytic route is consistent with dual-process theory (e.g., CEST) to the extent that appraisal-based processes (such as perception of risk) are viewed to mediate the relationship between effortful cognitive evaluation and behavioral outcomes (in this context terrorism-related behavior change) (Epstein et al., 1996).

### Limitations and Future Considerations

An important limitation within the present study was that measures were self-report and assessed only subjective evaluations of behavior change, risk and thinking style. In this context, it is unclear whether responses actually reflected real world intentions and behaviors. This problem is not unique to this particular piece of research, but reflects the fact that self-report measures provide only snapshots of processes and thoughts. In the case of risk perception, the relationship between threat and behavior is uncertain. People engage in many risky behaviors and correspondingly avoid many low risk activities. Similarly, there is variance between intentions to change behavior, actual behavior adaptation and adherence to change. In the context of reality testing, the IPO-RT assessed only subjective evaluation of perceived likelihood of reality testing errors. Due to the extemporaneous nature of reality testing decisions, individuals may lack conscious awareness, or insight into their veracity. This is likely to affect self-report measure accuracy. This is true of metacognitive measures generally. Accordingly, the relationship between subjective performance and actual performance is often weak.

As noted above, emotional reactions often bias thinking away from a cognitive-analytic style (e.g., Epstein et al., 1996). Particularly, emotions influence perception (e.g., Slovic et al., 2002; Lerner et al., 2003; Hogarth et al., 2011). The current model did not directly assess emotional reactions as either predictors or dependent responses to risk and terror situations. Hence, it is impossible within the present study to make conclusions regarding the role of emotions. Accordingly, subsequent research should investigate further how emotional reactions influence risk perception and terrorism-related behavior change.

The link between terrorism-related behavior change and a preference for subjective-intuitive thinking, as conceptualized within dual-process models, requires further examination. This is because the present study failed to measure directly the types of processing specified by dual-process models. Rather, the current investigation made use of measures that act as ‘proxies’ for these styles of thinking. Effectively, there is no *direct* evidence that the two factors measured here represent distinct processing modes. Nevertheless, the dual-process approach (i.e., CEST) provides a framework in which to assess the current findings, evaluate study limitations and specify the remit of subsequent research. From this perspective, future work on perception of risk and terrorism-related behavior change could align itself more closely with CEST and its measurement instruments (such as the Rational-Experiential Inventory, or the Rational/Experiential Multimodal Inventory). In addition to assessing individual differences, experimental analyses could be of value in determining the characteristics of different modes of thinking and the extent to which they underpin terrorism-related judgments of risk. For example, if judgments derive from intuitive modes of thinking, then these should be insensitive to manipulations that load working memory or attentional processes. Clearly, the current work, although setting the foundations for considering terrorism-related judgments of risk within a dual-process framework, needs to be more explicit in aligning and evaluating this contention.

Concern for personal well-being and avoidance of physical danger affects terrorism-related perception of risk and behavior change. This study considered only behavior adaption. Consequently, future research may wish to examine which specific terrorism-related risk perceptions best predict behavior change. Potential variables are risk attached to personal safety, especially injury or death. At a practical level, it is important to know which risk perceptions and behaviors are malleable and which are resistant to modification. From a counter-terrorism perspective, this information would help to advise strategy. Particularly, services and government policy could identify and target important, undervalued factors. This would help to facilitate and guide effective terrorism-related behavior change.

In the present study, the use of the term ‘previous terrorist attacks’ was rather vague and non-specific. Future work could improve event salience by referring to specific or more detailed events. Within these, systematic manipulation of factors, such as proximity, extent and outcome of attack would enable researchers to identify motivating features of behavior change. A further variable to consider is immediacy. Following high profile, terrorist attacks intention and motivation to change behavior are likely to be high. Charting intention to change and adherence to modification over time would also prove informative. Assessing important variables related to change (particularly extent, adherence and period of time during which adaptation is likely) could prove highly instructive.

Data in the present study was cross-sectional. Collecting data at one point in time is problematic because it is not possible to establish causal links within models. However, it is still permissible to make predictive inferences when specification of tested models is *a priori* and SEM is used (Hubbard and Mannell, 2001). In these circumstances, good data fit provides evidence for model legitimacy, facilitating inferences about relationships (Bollen, 1989). This article met these criteria.

This work has important real world applications. Particularly, it suggests that perception of risk-related events (i.e., media portrayals) links to behavior change. This notion is consistent with earlier work on social amplification of risk, which proposes an interaction between risk events and social processes (see Kasperson et al., 1988). In this context, risk *per se* does not exist and distinctions between true (absolute) and distorted (socially determined) are redundant. Instead, characteristics of public response arising from institutional information and social amplification determine perceptions of risk (Kasperson et al., 1988). In part, this explains why people possess distorted perceptions of terrorism-related threat. An extreme example of this is terrorism focused moral panics. As Rothe and Muzzatti (2004) report, media and political depictions of terrorists and terrorism have contributed to excessive levels of panic and fear, misguided public consciousness, and the development of social detrimental legislation. The low levels of observed terrorism-related behavior change within this paper do not diminish the importance of the findings because minor changes in public behavior can produce direct and indirect (ripple) effects, which have major societal consequences (Kasperson et al., 1988; Everard et al., 2016). For instance, increased driving following the 9/11 attacks resulted in increased road traffic fatalities (Blalock et al., 2009). Future research should examine this further.

## Author Contributions

AD: theoretical focus, data analysis, and article development. ND: theoretical focus, data analysis, and article development. KD: contributed to the writing process. AP: contributed to the writing process. PC: advised on paper and assisted with drafting.

## Conflict of Interest Statement

The authors declare that the research was conducted in the absence of any commercial or financial relationships that could be construed as a potential conflict of interest. The reviewer KS and handling Editor declared their shared affiliation.
