# Supplementary figures and images for: Perception of Risk and Terrorism-Related Behavior Change: Dual Influences of Probabilistic Reasoning and Reality Testing
Source: Front Psychol. 2017 Oct 5;8:1721. doi: 10.3389/fpsyg.2017.01721 (PMC5633603; doi:10.3389/fpsyg.2017.01721)

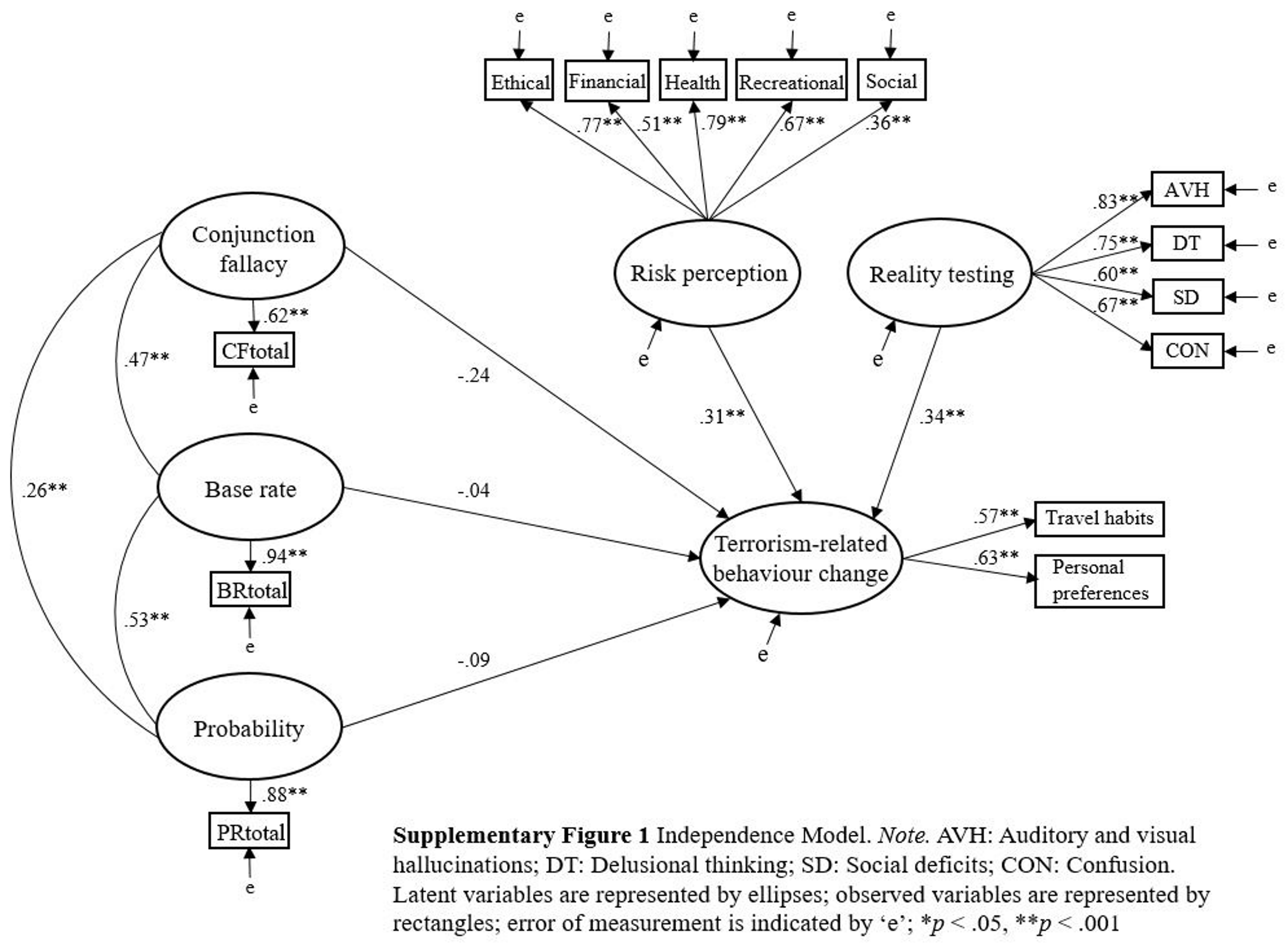

Supplement: Supplementary file 1 [file Image_1.TIF]

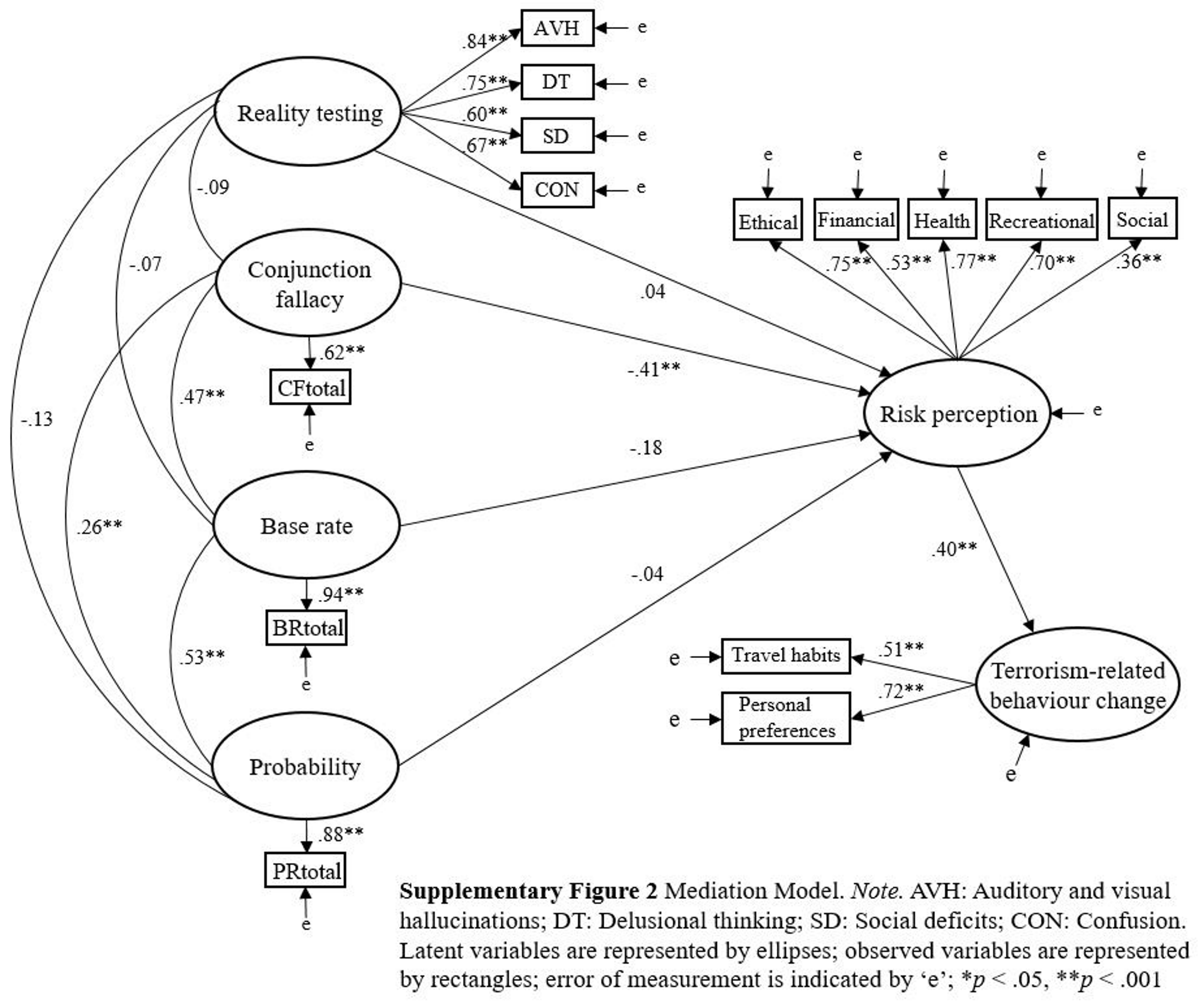

Supplement: Supplementary file 2 [file Image_2.TIF]
